# Prevalence and Risk Factors of Occult HCV Infection in the Adult Population of Mexico City

**DOI:** 10.3390/v17020236

**Published:** 2025-02-08

**Authors:** Luis Antonio Uribe-Noguez, María Erandhi Prieto-Torres, Luis Octavio Uribe-Noguez, José Antonio Mata-Marín, Carla Ileana Arroyo-Anduiza, Rebeca Paquentín-Jimenez, Alberto Chaparro-Sanchez, Wendy Guadalupe Vazquez-Gonzalez, Andrea Santos Coy-Arechavaleta, Ericka Nelly Pompa-Mera, Jesus Gaytán-Martínez, Julio Elias Alvarado-Yaah, Clara Esperanza Santacruz-Tinoco, Alicia Ocaña-Mondragón

**Affiliations:** 1 Laboratorio Central de Epidemiología, División de Laboratorios Especializados, Centro Médico Nacional (CMN) “La Raza”, IMSS, México City 02990, Mexico; wendisya@live.com.mx (W.G.V.-G.); santoscoycita@gmail.com (A.S.C.-A.); alvaram25@outlook.com (J.E.A.-Y.); oama14@yahoo.com.mx (A.O.-M.); 2 Coordinación de Información y Análisis Estratégico, Instituto Mexicano del Seguro Social (IMSS), Mexico City 77503, Mexico; eran24dias@hotmail.com; 3 Salud Digna, Mexico City 55717, Mexico; siuluribe@hotmail.com; 4 Departamento de Enfermedades Infecciosas, Hospital de Infectología, CMN “La Raza”, IMSS, Mexico City 02990, Mexico; jamatamarin@gmail.com (J.A.M.-M.); a_chaparro@hotmail.com (A.C.-S.); jgaytanmtz@yahoo.com.mx (J.G.-M.); 5 Departamento de Patología Clínica, Banco Central de Sangre, CMN “La Raza”, IMSS, México City 02990, Mexico; carlaileana@gmail.com; 6 Inflammatory Eye Disease Clinic, Asociación Para Evitar la Ceguera en México, Hospital “Dr. Luis Sánchez Bulnes”, México City 04030, Mexico; rebecapj29@gmail.com; 7 Unidad de Investigación en Enfermedades Infecciosas y Parasitarias, Hospital de Pediatría, CMN “Siglo XXI”, IMSS, Mexico City 06720, Mexico; erickanelly@yahoo.com.mx; 8 División de Laboratorios Especializados, Instituto Mexicano del Seguro Social, Mexico City 07760, Mexico; clara.santacruz@imss.gob.mx

**Keywords:** HCV, occult HCV infection, general population, risk factors, sequencing

## Abstract

Occult HCV infection (OCI) is defined by the presence of HCV RNA in hepatocytes and/or peripheral blood mononuclear cells (PBMCs) without detectable HCV RNA or anti-HCV antibodies in plasma. OCI is underrecognized and may contribute to HCV transmission. This study estimated OCI prevalence and associated risk factors in adults from Mexico City. **Methods**: A retrospective cross-sectional study was conducted, analyzing 507 general population volunteers. Demographic data and potential risk factors were collected via questionnaire. Anti-HCV detection was performed using two techniques: immunochromatographic rapid test and chemiluminescent microparticle immunoassay (CMIA). Nested PCR was employed to detect HCV RNA in plasma and PBMCs. Positive samples were genotyped through sequencing and phylogenetic analysis of the *Core/E1* region. **Results**: Of 507 participants, four were anti-HCV positive. HCV RNA was found in PBMCs of 27 individuals, while plasma samples tested negative, indicating a 5.3% OCI prevalence. OCI was significantly associated with blood donation (*p* = 0.015), drug use (*p* = 0.019), particularly cocaine (*p* = 0.001), and endoscopy (*p* = 0.043). Genotypes 1b, 1a, 2b, 3a, and 2j were detected in OCI cases. **Conclusions**: OCI prevalence in Mexico City’s general population is notable, with significant links to blood donation, cocaine use, and endoscopy. Enhanced diagnostic strategies are crucial to detect OCI and mitigate HCV transmission.

## 1. Introduction

The hepatitis C virus (HCV) belongs to the *Flaviviridae* family, genus *Hepacivirus*. Its genome corresponds to positive-sense, single-stranded RNA, of approximately 9.6 KB [1]. Globally, about 50 million people are infected with HCV, and 242,000 people die annually from related causes [2]. In Mexico, between 400,000 and 1.4 million people are estimated to be serologically positive for HCV and about 530,000 of these individuals show chronic replicative infection and require antiviral treatment. The primary causes of HCV infection in Mexico include blood transfusion, surgery, and sexual practices involving blood exposure [2,3,4,5].

New antiviral therapies have shown improved sustained viral response (SVR), defined as the absence of detectable HCV RNA in the blood 12 weeks or more after completing antiviral therapy. Consequently, the WHO has recognized SVR as a critical pathway to eradicating HCV infection, aiming to mitigate the disease’s threat to global public health by 2030 [6,7]. Accordingly, the WHO has established strategies to reach a reduction in new infections by 90% and deaths by 65% between 2016 and 2030. Currently, access to and acceptance of HCV diagnostic tests remain low. Increasing the availability of quantitative tests is essential for diagnosing more cases, initiating treatments, and achieving SVR, thereby aligning with the WHO’s goals [6,7,8,9].

Diagnosis and monitoring of HCV infection are based in specific antibody testing and HCV RNA detection in plasma or serum [10,11]. However, HCV’s ability to replicate in peripheral blood mononuclear cells (PBMCs) is evidenced by the detection of the HCV RNA negative strand, which might lead to underestimating HCV prevalence and facilitating its spread [12,13]. In 2004, Castillo et al. reported the presence of occult HCV infection (OCI) in patients with chronic hepatitis of unknown etiology. OCI is characterized by (1) detection of HCV RNA in liver tissue alone or in liver tissue and/or PBMCs, and (2) consistently undetectable HCV RNA in plasma or serum by routine laboratory and blood bank detection methods, with or without transaminase elevation [14,15]. There are two types of OCI: seronegative OCI (negative for anti-HCV and plasma/serum HCV RNA) and seropositive OCI (anti-HCV positive but plasma/serum HCV RNA negative), also called secondary occult HCV infection. This study focuses on seronegative OCI [14,16,17,18].

Previous studies have reported OCI prevalence in various groups, such as subjects with cryptogenic hepatic disease (0 to 74.2%) [19,20], patients undergoing hemodialysis (0.25 to 45%), and blood donors (2.1 to 5.7%) [19,20,21,22,23]. In Mexico, OCI prevalence among blood donors is 3.4% [23]. De Marco et al. reported an OCI prevalence of 3.3% and 1.27% in an infectious liver disease-free (ILDF) population, who also had normal-range hepatic enzymes and did not have serological markers for HCV infection [24,25]. Although evidence of OCI in risk groups has been reported, there is still major uncertainty regarding optimal diagnostic approaches and population prioritization for diagnostic testing [8]. OCI can notably occur in the general population without apparent symptoms, posing a potential risk for HCV transmission [14,15]. Current reference laboratory and blood bank techniques do not include HCV RNA detection in PBMCs [10], leaving OCI prevalence in the general population largely unknown. This study aims to determine the prevalence and risk factors of OCI in the general adult population of Mexico City.

## 2. Materials and Methods

### 2.1. Study Population

In this retrospective cross-sectional study, 550 volunteers from the general population were recruited in the area surrounding the Central Blood Bank of the National Medical Center “La Raza” of IMSS. The subjects were recruited from among individuals circulating in the streets surrounding the Central Blood Bank; they were not blood donors or individuals attending the hospital. These individuals were not simply passersby nor people experiencing homelessness, but rather volunteers from the general population who were circulating near the Central Blood Bank. They were directly invited by study personnel following a brief explanation of the study objectives. Recruitment included individuals who met specific inclusion criteria: being 18 years of age or older, possessing a valid official identification. These volunteers self-declared being free from any ILDF and provided informed consent. They also self-reported as “healthy” against any infectious liver disease and reported no history of previous hepatic infections or receiving a previous antiviral treatment for HCV or HBV. All voluntarily accepted to participate in this study by signing a written informed consent. Each participant completed a specifically designed questionnaire to collect demographic data and detect OCI risk factors (Supplementary Document S1). The study followed the guidelines of the Strengthening the Reporting of Observational Studies in Epidemiology (STROBE) criteria 2.2. Plasma and PBMCs isolation

Peripheral blood samples were obtained via venous puncture and collected in tubes with EDTA (Becton, Dickinson and Company, Franklin Lakes, NJ, USA). Plasma was separated by centrifugation for 15 min at 3000 RPM at room temperature. PBMCs were isolated by following the methodology of Martínez-Rodríguez et al. [23]. PBMCs were resuspended in 250 µL of TRIzol™ Reagent (Invitrogen Life Technologies, Burlington, ON, Canada), and the storage and handling of plasma and PBMCs were carefully controlled. All of these materials, including centrifuged plasma and isolated PBMCs, were stored at −80 °C immediately after blood sampling and analyzed without cycles of freezing and thawing.

### 2.2. HCV Serology and Laboratory Tests

Anti-HCV detection was made by two techniques: immunochromatographic rapid test “SD BIOLINE HCV” (Standard Diagnostics Inc., Yongin, Gyeonggi-do, Republic of Korea) following the manufacturer’s instructions, and by chemiluminescent microparticle immunoassay (CMIA) in the equipment ARCHITECT i2000SR (Abbott, Abbott Park, IL, USA) using the ARCHITECT Anti-HCV Reagent Kit (Abbott, Abbott Park, IL, USA) following manufacturer’s instructions. The sensitivity and specificity of both methods are >98% [26]. Detection of antibodies against hepatitis B Virus (HBV), human immunodeficiency virus (HIV), *Treponema pallidum*, and *Trypanosoma cruzi* was performed by CMIA in the equipment ARCHITECT i2000SR (Abbott, Abbott Park, IL, USA) following the manufacturer’s instructions.

### 2.3. Transaminase and Bilirubin Detection

The levels of alanine aminotransferase (ALT), aspartate aminotransferase (AST), direct bilirubin (DB), and total bilirubin (TB) were measured using the automated equipment Dimension RxLMax (Siemens Healthcare Diagnostic Inc., Newark, NJ, USA) following the manufacturer’s instructions. The following reference values were used: ALT < 55 UI/L, AST < 37 IU/L, TB < 1 mg/dL, and DB < 0.3 mg/dL.

### 2.4. RNA Extraction and Reverse Transcription

Viral RNA from plasma was isolated using the QIAamp Viral RNA Mini Kit (QIAGEN, Hilden, Germany) and total RNA from PBMCs in TRIzol™ Reagent with the RNAeasy kit (QIAGEN Hilden Germany), following the manufacturer’s instructions. RNA from plasma and PBMCs was quantified on a NanoDrop 2000 (Thermo Fisher Scientific, Waltham, MA, USA) and adjusted to a 20 ng/µL concentration. The cDNA was obtained by reverse transcription using the AMV reverse transcriptase kit and random hexamer primers (Promega, Madison, WI, USA), following the manufacturer’s instructions. PBMCs and plasma from patients with confirmed chronic HCV infection and HCV-negative individuals were included as internal controls. The housekeeping gene of *β-actin* was amplified as an internal control of PCR both in the cells and in the plasma of each volunteer. The primers used were 5′-ATGTGGCCGAGGACTTTGATT-3′ and 5′-AGTGGGGTGGCTTTTAGGATG-3′. Amplification conditions consisted of an initial denaturation at 95 °C for 1 min, followed by 30 cycles of 95 °C for 15 s, 60 °C for 20 s, 72 °C for 15 s, and final extension at 72 °C for 5 min. A product of PCR of 110 bp indicated an adequate isolation of RNA and PCR analysis.

### 2.5. HCV Nested PCR

HCV RNA detection was performed by nested PCR using the MyTaq DNA Polymerase kit (Bioline, London, UK). The HCV genome region 5′UTR was amplified using previously reported primers [27,28]: fap: 5′-GCAGAAGCGTCTAGCCATGG-3′ and zap: 5′-CTCGCAAGCACCCTATCAGGC-3′ for the first amplification, and fip: 5′-TCTAGCCATGGCGTTAGTA-3′ and zip: 5′-CAGTACCACAAGGCCTTTC-3′ for the second amplification. The nested PCR program consisted of an initial denaturation at 95 °C for 1 min, followed by 28 cycles of 95 °C for 15 s, alignment at 55 °C for 20 s, extension at 72 °C for 15 s, and final extension at 72 °C for 10 min. PCR products, including negative and positive controls, and a 100 bp DNA ladder, were visualized by 1.5% agarose gel electrophoresis and stained with SYBR Green. The presence of an amplicon of 240 bp from the 5`UTR region, corresponding to nucleotide numbering of 80–320 in the H77 reference HCV genome (GenBank accession no. AF009606) was considered a positive test for RNA-HCV. With our system, we observed a sensitivity of approximately 50 genomic equivalents (6 IU/mL) [23].

### 2.6. Sequencing of Core/E1 HCV Region

A fragment of 473 bp from the *Core/E1* region, corresponding to nucleotide numbering of 843–1315 in the H77 reference HCV genome, was reverse-transcribed and amplified by semi-nested PCR using the MyTaq DNA Polymerase kit (Bioline, London, UK) and previously reported primers were used [29]: E1ExA: 5′-GTRGGNGACCARTTCATCATCA-3′ and E1ExS: 5′-GCAACAGGGAAYYTDCCYGGTTGCTC-3′ for the first amplification. The PCR conditions were as follows: initial denaturation at 95 °C for 1 min followed by 28 cycles of denaturation at 95 °C for 15 s, annealing at 56 °C for 20 s, and an extension at 72 °C for 15 s, followed by a final extension for 10 min. In the second round of amplification, the initiator E1InA: 5′-TTCATCATCATRTCCCANGCCAT-3′ and E1InS: 5′-AAYYTDCCCGGTTGCTCTTTYTCTAT-3′ were used for nested PCR, with the same PCR program. The amplicon was purified with a QIAquick Gel Extraction Kit (QIAGEN, Hilden, Germany), following the manufacturer’s instructions. DNA sequencing was performed, using the same primers as for the nested PCR, at the Molecular Biology Unit, In-stituto de Fisiología Celular, Universidad Autónoma de México, Mexico City, using a 3500 Genetic Analyzer with the BigDye Terminator v3.1 Cycle Sequencing kit (Thermo Fisher Scientific, Waltham, MA, USA).

### 2.7. Determination of HCV Genotypes

The *Core/E1* region of HCV RNA from the 5′-UTR PCR-positive samples was amplified by RT-PCR for HCV genotype determination. Nucleotide sequences obtained in region *Core/E1* were manually edited with BioEdit v7.0.9.0 (Ibis Biosciences, Raleigh, NC, USA) [30] and were aligned by ClustalW (European Bioinformatics Institute, Hinxton, Cambridgeshire, UK) [31] with reference sequences representing HCV genotypes and subtypes retrieved from GenBank [32]. Phylogenetic trees were further constructed in MEGA v7 software (Arizona State University, Tempe, AZ, USA) using the maximum likelihood (ML) method with the GTR + G + I model for computing evolutionary distances [33]. Robustness was estimated by bootstrap analysis with 1000 pseudo-replicate data sets, and only bootstrap values > 70 were considered significant. All sequences were subjected to confirmation with the Genotyping tool of the National Center for Biotechnology Information (NCBI, NCBI Genotyping Tool, National Institutes of Health, Bethesda, MD, USA) [34].

### 2.8. Statistical Analysis

All data were tabulated and processed using the Statistical Package for the Social Sciences program (SPSS Statistics v21.0, IBM, Armonk, NY, USA). The categorical and discrete variables were analyzed with the Chi squared *X^2^* and the exact Fisher test. The risk factor strength of association was estimated using an odds ratio (OR) with a confidence interval of 95%. The differences were considered statistically significant with a *p* value <0.05. Independent risk factors associated with OCI transmission were identified in the logistic regression analysis (calculated with a confidence interval of 95%), which included the significant variables (*p* < 0.05) from the bivariate analysis.

## 3. Results

### 3.1. Demographic Characteristics, Serology, and Laboratory Tests

Initially, 550 subjects were recruited between July 2018 and April 2019. Forty-three participants were excluded for various reasons: 12 refused to complete the questionnaire, 13 had insufficient blood samples, and 18 had incomplete questionnaires or medical records. The final study population consisted of 507 subjects, of which 314 were men and 193 were women. Four patients with specific antibodies against HCV were identified using both the rapid test “SD BIOLINE HCV” and CMIA, with concordant results between the two methodologies. However, these patients were negative for HCV RNA in PBMCs or plasma, indicating spontaneous hepatitis C viral clearance. Five participants were identified as having other comorbidities: three were positive for HIV by serology, one was positive for anti-HBV, and one was positive for *Trypanosoma cruzi*, with no participants testing positive for *Treponema pallidum*. None of these individuals presented coinfection with HCV. These participants were promptly notified of the findings. The principal age range was 30 to 39 years old (*n* = 170), followed by 18 to 29 years old (*n* = 167). In the transaminase and bilirubin evaluations, elevated levels of ALT (>55 IU/L) were found in 73 subjects, elevated levels of AST (>37 IU/L) were found in 39 subjects, elevated levels of total bilirubin (>1 mg/dL) were found in 64 subjects, and elevated levels of direct bilirubin (>0.3 mg/dL) in 12 subjects.

Among the participants, 27 subjects (15 men and 12 women) tested positive for HCV RNA in PBMCs but negative in plasma. The prevalence of OCI in the studied population was 5.3%. There was no significant difference in OCI prevalence between genders (OR: 1.3; 95% CI; 0.61–2.87; *p* = 0.306). OCI was most frequent in subjects aged 30 to 39 years, showing a significant difference (OR: 2.23; 95% CI; 1.03–4.87; *p* = 0.034). No significant association was found between the presence of OCI and elevated ALT (OR: 4.63; 95% CI; 0.6–34.34; *p* = 0.078), AST (OR: 2.24; 95% CI; 0.3–17.0; *p* = 0.367), or TB (OR: 0.82; 95% CI; 0.28–2.48; *p* = 0.451). DB elevated values were not found in subjects with OCI. Results are shown in Table 1.

### 3.2. Risk Factors

OCI was significantly associated with drug abuse (OR: 2.94; 95% CI: 1.23–7.03; *p* = 0.019), particularly cocaine consumption (OR: 6.93; 95% CI: 2.50–19.16; *p* = 0.001). Undergoing an endoscopy was also significantly related (*p* = 0.043). No association was found between OCI and sexual preference, previous transfusions, surgeries, tattoos, piercings, dental procedures, or incarceration (Table 2).

A logistic regression model identified cocaine consumption (OR: 6.313; 95% CI: 1.15–34.59; *p* = 0.034) and undergoing an endoscopy (OR: 3.224; 95% CI: 1.11–9.36; *p* = 0.031) as statistically significant factors (Table 3).

### 3.3. Determination of the HCV Genotype by Sequence Analysis

**Figure 1:**
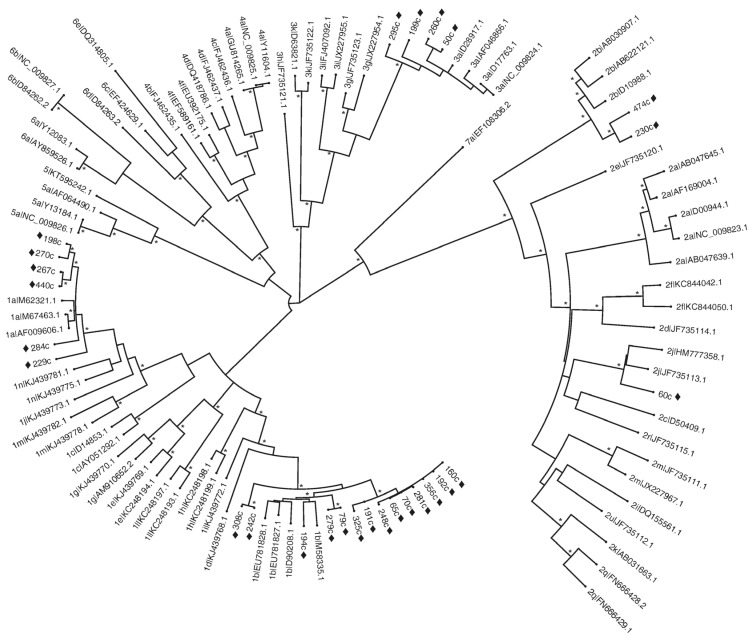
Maximum likelihood phylogenetic tree of the *Core/E1* region. The diagram shows esti-mated maximum likelihood phylogeny of reference and study-generated sub-genomic *Core/E1* sequences. Identified 27 samples (52%) as GT1b, six (22%) as GT1a, and four (15%) as GT3a. Among the GT2 subtypes, two samples (7%) were identified as subtype 2b and one (4%) as GT2j. Sequences generated in this study are prefixed with “♦”. * Indicates bootstrap values of >70% at the beginning of the roots corresponding to cluster. Reference sequences are labeled with their subtypes and accession numbers. The tree was constructed using MEGA v.7 software, by the maximum likelihood method. The tree was inferred by the GTR + G + I model for nucleotide substitutions. Sequences obtained were stored in GenBank: *Core/E1* Region (GenBank accession no. MZ997434 to MZ997460).

Phylogenetic analysis grouped OCI sample sequences into five clusters corresponding to GT1a, GT1b, GT2b, GT2j, and GT3a, in comparison with the reference sequences (Figure 1). The analysis showed identical subtyping to the NCBI Genotyping analysis, with strong bootstrap supports >85% (Supplementary Table S1). Fourteen OCI-positive samples (52%) were identified as GT1b, six (22%) as GT1a, and four (15%) as GT3a. Among the GT2 subtypes, two samples (7%) were identified as subtype 2b and one (4%) as GT2j. GenBank access codes are listed in Supplementary Table S1.

## 4. Discussion

In the present study, we analyzed 507 general population volunteers located around the Central Blood Bank of the National Medical Center “La Raza” of IMSS. These volunteers self-declared as free from any ILDF and provided informed consent. We identified OCI in 27 subjects (15 men and 12 women), reporting a prevalence of 5.3%. The most common age range for OCI was 30–39 years. There was not a significant association between the presence of OCI and elevated levels of ALT, AST, DB, or TB. Our results confirmed previous reports that OCI is significantly associated with drug abuse, specifically cocaine use [35,36], and undergoing endoscopic procedures. While high-level disinfection of endoscopes is standard, HCV transmission through these procedures, although rare, has been documented [37,38,39]. Conversely, other known HCV risk factors such as sexual preference, previous transfusions, surgeries, tattoos, piercings, dental procedures, and incarceration were not found to be risk factors in this study. Genotyping revealed the presence of HCV genotypes 1b, 1a, 2b, 3a, and the rare 2j subtype among OCI-positive individuals. These findings are consistent with the distribution of HCV genotypes in Mexico [40].

This study highlights the diversity of genotypes in OCI infections within a population from Mexico City, identifying GT1a, GT1b, GT2b, GT2j, and GT3a, which underscores the genetic heterogeneity of HCV in OCI patients [17,19,21,41]. The presence of less common subtypes, such as GT2j, suggests that specific viral variants with distinct cellular tropisms may contribute to their persistence in extracirculatory compartments, such as PBMCs [11,42,43]. Moreover, the detection of GT2j reinforces the notion that migration dynamics and globalization are driving the emergence of less common subtypes in different regions [44,45,46]. These findings are relevant to the molecular epidemiology of HCV and OCI, as genotypes are key factors predicting sustained virological response (SVR). However, it remains unknown whether extrahepatic sites, which have reduced rates of viral replication and exhibit extremely low viral loads, are less susceptible to DAA treatment [11,12].

Routine tests for HCV infection diagnosis include the specific anti-HCV antibodies via ELISA and RNA detection in serum or plasma. In our study, four subjects were anti-HCV positive but HCV RNA negative in PBMCs or plasma, likely indicating spontaneous clearance of acute infection [47]. This highlights the limitation of conventional serological tests, which do not detect OCI, an underrecognized form of HCV infection.

The type of patient sample analyzed has a significant impact on OCI detection rates. Liver biopsies are considered the gold standard for OCI diagnosis [48]. However, due to the limited availability of this material, OCI is often detected in plasma, serum, and/or PBMCs [16,48]. The use of PBMCs instead of liver biopsies in the present study is justified for several ethical, practical, and scientific reasons. Firstly, liver biopsies are invasive procedures that carry risks for patients, such as bleeding, infection, and pain. Since this study was conducted in a general volunteer population, subjecting participants to such an invasive procedure would be unethical, particularly when no clinical indication justifies it. Regarding the functional equivalence between PBMCs and liver tissue, various studies have reported that PBMCs can reflect the presence of HCV in extracirculatory compartments [11,17,19,49]. For instance, research has found that PBMCs can act as reservoirs of viral RNA, making them a valid source for studying OCI in the absence of liver biopsies [14,24,25,50,51,52].

Our study found an OCI prevalence of 5.3% in the general population, significantly higher than the 0.6% prevalence of active viremia reported in Mexico [53]. This discrepancy suggests substantial underestimation of OCI and highlights its potential role in HCV transmission. De Marco et al. studied the presence of OCI in healthy subjects that did not have positive indicators for hepatic disease, obtaining similar prevalence results (3.3% and 1.27%); however, risk factors for OCI were not determined in these studies [24,25].

Although some studies report elevated hepatic enzyme activity in OCI patients [14,54], in this study, no significant association was found between the presence of OCI and elevated levels of ALT (*p* = 0.078) or AST (*p* = 0.367). Only one volunteer with OCI (3.7%) had elevated AST and ALT. These findings may suggest that volunteers could have been in an early stage of infection and/or that liver damage in OCI progresses more slowly than in typical HCV infection. This information aligns with the data reported by Esaki et al. and Comar et al., who concluded that OCI seems to be more benign than HCV infection; nevertheless, it can still potentially progress to hepatic cirrhosis and hepatocellular carcinoma [55,56].

We did not find a significant association between OCI and gender. However, OCI was more common in the 30–39 age group (*p* = 0.034), suggesting progression from asymptomatic to chronic infection over decades, similar to chronic HCV infection’s natural history [57]. A study described that 81% of the subjects who were positive for HCV RNA had donated blood in the last 5 years, which explains the significant presence of OCI in the blood donor population in Mexico City [23]. This highlights the need for more sensitive detection methods in blood banks and reference laboratories, as OCI may contribute to ongoing HCV transmission in these settings.

In this study, a significant relationship was found between OCI and drug abuse (24% of people using cocaine were positive for OCI). Cocaine use emerged as a significant risk factor for OCI, with an OR of 6.8, indicating a higher infection risk compared to chronic HCV infection. In Mexico, the most common drugs are cannabis and cocaine, while injectable drugs are consumed by a very small segment of the population [58]. This aligns with previous studies showing increased HCV infection risk with non-injectable drug use. In 2013, Sugden et al. studied 20 intravenous drug users and reported that two (10%) of these subjects were positive for OCI, as evidenced by HCV RNA in their PBMCs [59]. Macías et al. reported an HCV infection prevalence of 12.6% in non-intravenous drug users, with sharing cocaine inhalation straws yielding an OR of 3.6 [60]. The significant association of OCI with endoscopies (*p* = 0.043) raises concerns about potential lapses in infection control practices during these procedures [61,62]. Although modern endoscopic techniques generally adhere to strict sterilization protocols, our findings suggest that transmission risks may persist and warrant further investigation and improvement of preventive measures.

Although blood transfusion, surgeries, piercings, tattoos, and incarceration did not show associations with OCI in this study, they remain significant risk factors for HCV and should not be overlooked. A study published by Miners et al. reported beneficial results regarding cost terms and epidemiological descriptions of HCV by performing diagnostic tests in migrant populations entering England [63]. Kileng et al. reported that even in regions with very low prevalence, the current strategy of screening only individuals with a high risk of infection may be an inadequate approach to identify chronic HCV infection hidden within the general population [64]. The inclusion of HCV and OCI screening strategies in the general population represents not only an opportunity to recognize HCV prevalence, but also to assess the clinical and economic burden while defining the most effective screening program for this disease [65]. Expanding HCV and OCI screening to the general population could improve epidemiological control and reduce the public health burden.

Current estimates suggest that fewer than 20% of HCV-infected individuals are aware of their condition, excluding OCI cases. Our findings underscore the epidemiological significance of OCI and highlight the need to expand access to diagnostic tests for both HCV and OCI to meet the WHO’s elimination goals [5,66,67]. Previous studies have primarily focused on high-risk groups, such as polytransfused patients, those with hepatic disease of unknown etiology, HIV co-infected patients, and individuals undergoing hemodialysis [13,17,19,68,69,70]. We encourage considering viral hepatitis molecular diagnostic tests be done as routine tests for the general population, and/or specifically aimed at the most affected or high-risk populations. OCI’s asymptomatic nature and the inadequacy of current detection methods contribute to the silent spread of HCV [64,65].

While the optimal treatment for OCI remains unclear, exploring the efficacy of direct-acting antivirals (DAAs) for OCI patients could provide new therapeutic avenues [17]. Our study’s findings contribute to understanding OCI’s prevalence and risk factors, emphasizing the need for enhanced diagnostic and screening strategies to support global HCV elimination efforts.

The present study has several limitations that should be considered when interpreting its results. First, the retrospective design entails inherent risks of selection bias, which may affect the representativeness of the sample and the generalizability of the findings. Additionally, although 507 participants were included, the relatively small number of positive OCI cases (27 individuals) further limits the extrapolation of the results. The use of PBMCs instead of liver biopsies, although justified for ethical and practical reasons, may have reduced sensitivity in detecting OCI, given that liver biopsies are considered the gold standard. Furthermore, reliance on self-reported data for risk factors such as drug use introduces recall and social desirability biases. It is highly likely that the prevalence of OCI may increase, for example, among older generations due to the lack of safe sexual practices that younger generations are more likely to adopt. The lack of longitudinal follow-up prevents the assessment of OCI clinical progression, while the geographically localized nature of the sample may not be representative of other regions or sociodemographic groups. Finally, although less common genotypes were identified, the limited sample size may not accurately reflect the true genotypic diversity of HCV in the population. These limitations highlight the need for further research with prospective designs, larger cohorts, and more sensitive diagnostic tools to address these constraints and validate the current findings.

## 5. Conclusions

In conclusion, this study is the first in Mexico to establish OCI prevalence in the general population, identifying significant risk factors such as cocaine use and endoscopic procedures. Addressing OCI detection is essential for achieving HCV elimination. Further studies with larger sample sizes are needed to better understand OCI’s epidemiology and its role in HCV transmission.

## Figures and Tables

**Table 1:** Demographic characteristics, transaminase, and bilirubin serum levels of studied population.

Category	Total	OCI (+)	X2/Fisher	Odds Ratio	95% CI	*p*-Value
**Gender**			0.492			
Male	314	15		1.0	Referent	-
Female	193	12		1.3	0.61–2.87	0.306
**Age**			0.093			
18–29	169	5		0.43	0.16–1.18	0.067
30–39	170	14		2.23	1.027–4.87	0.034 *
40–49	122	7		1.11	0.45–2.69	0.485
50–59	39	0		1.06	1.03–1.08	0.128
>60	7	1		3.03	0.35–26.17	0.320
**ALT**			0.104			
Normal	434	26		1.0	Referent	-
Elevated	73	1		4.63	0.6–34.34	0.078
**AST**			0.424			
Normal	468	26		1.0	Referent	-
Elevated	39	1		2.24	0.3–17.0	0.367
**TB**			0.725			
Normal	443	23		1.0	Referent	-
Elevated	64	4		0.82	0.28–2.48	0.451
**DB**			0.406			
Normal	495	27		1.0	Referent	-
Elevated	12	0		N/A	N/A	N/A
**Previous blood donations**			0.021 *			
No	201	5		1.00	Referent	-
Yes	306	22		3.05	1.13–8.15	0.015 *

OCI: occult hepatitis C virus infection; ALT: alanine aminotransferase; AST: aspartate aminotransferase; TB: total bilirubin; DB: direct bilirubin; X2: Pearson chi-square test; F: Fisher exact test; * Indicates a significant result (*p* < 0.05, two-tailed).

**Table 2:** Risk factors associated with occult HCV infection.

Risk Factor	Total	OCI (+)	X2/Fisher	OR	95% CI	*p*-Value
**Sexual Preference**			0.917			
Heterosexual	490	26		1.00	Referent	-
Homosexual	17	1		0.89	0.11–7.02	0.612
Bisexual	0	0		N/A	N/A	N/A
**Transfusions**			0.814			
No	492	26		1.00	Referent	-
Yes	15	1		1.28	0.16–10.1	0.565
**Surgery**			0.330			
No	326	15		1.00	Referent	-
Yes	181	12		1.47	0.67–3.21	0.219
**Used Drugs** ^1^			0.011 *			
No	439	19		1.00	Referent	-
Yes	68	8		2.94	1.23–7.03	0.019 *
**Marijuana user**			0.235			
No	449	22		1.00	Referent	-
Yes	58	5		1.83	0.66–5.03	0.185
**Cocaine user**			<0.001 *			
No	482	21		1.00	Referent	-
Yes	25	6		6.93	2.50–19.16	0.001 *
**Tattoos**			0.865			
No	461	25		1.00	Referent	-
Yes	42	2		0.88	0.20–3.85	0.609
**Piercing**			0.417			
No	444	25		1.00	Referent	-
Yes	63	2		0.55	0.13–2.38	0.324
**Acupuncture**			0.375			
No	465	26		1.00	Referent	-
Yes	42	1		0.412	0.05–3.11	0.325
**Incarcerated**			0.499			
No	499	27		1.00	Referent	-
Yes	8	0		1.06	1.04–1.08	0.643
**Dental Surgery**			0.894			
No	175	9		1.00	Referent	-
Yes	332	18		1.05	0.46–2.40	0.538

OCI: occult hepatitis C virus infection; X2: Pearson chi-square test; F: Fisher exact test; * Indicates a significant result (*p* < 0.05); ^1^ The term ’Drug Use’ includes the consumption of any drug, regardless of its route of administration, and specifically includes the drugs mentioned separately (cocaine and marijuana).

**Table 3:** Multivariate analysis.

Risk Factors	aOR	95% CI	*p*-Value
Cocaine user	6.313	1.15–34.59	0.034 *
Endoscopy	3.224	1.11–9.36	0.031 *

aOR: adjusted odds ratio; 95% CI: 95% confidence interval; * Indicates a significant result (*p* < 0.05).

## Data Availability

The data that support the findings of this study are available on request from the corresponding author (L.A.U.N). The data are not publicly available due to privacy or ethical restrictions.

## Supplementary Materials

The following supporting information can be downloaded at https://www.mdpi.com/article/10.3390/v17020236/s1: Supplementary Document S1. Data collection sheet; Supplementary Table S1. Detailed genotyping results and patient characteristics.

## Institutional Revive Board Statement

The protocol was approved by the IMSS local research committee with the registration number R-2013-3502-59. All participants involved in this study provided their informed consent to take part in the study, which was conducted in accordance with the Declaration of Helsinki. The ethics committee applied specific restrictions for this study when publicly sharing de-identified data sets, as this information is considered by the committee to be potentially sensitive information.

## Abbreviations

ALT	Alanine Aminotransferase
AST	Aspartate Aminotransferase
CMIA	Chemiluminescent Microparticle Immunoassay
DAAs	Direct-Acting Antivirals
DB	Direct Bilirubin
GT	Genotype
HCV	Hepatitis C Virus
ILDF	Infectious Liver Disease-Free
NCBI	National Center for Biotechnology Information
OCI	Occult HCV Infection
OR	Odds Ratio
PBMCs	Peripheral Blood Mononuclear Cells
SVR	Sustained Viral Response
TB	Total Bilirubin
