# Supplementary material for: Prevalence and Risk Factors of Occult HCV Infection in the Adult Population of Mexico City
Source: Viruses. 2025 Feb 8;17(2):236. doi: 10.3390/v17020236 (PMC11860181; doi:10.3390/v17020236)
Supplement: Supplementary file 1 [file viruses-17-00236-s001.zip › Data collection sheet.pdf]

Data Collection Sheet

Prevalence of Occult Hepatitis C Virus Infection in the General Population of Mexico City

Record Number: _____________

All the information provided is STRICTLY CONFIDENTIAL and will only be used for statistical purposes. No results from this study will refer to specific individuals.

Instructions:
Please read the following questionnaire carefully, circle or cross out the appropriate response, or fill in the required spaces.

If you have any questions, feel free to ask. Thank you for your cooperation!

Gender:

☐ MALE ☐ FEMALE

Age (in years): _____________

Due to your occupation, are you or have you been in contact with biological-infectious material?

☐ YES ☐ NO

If the previous question is affirmative, have you been injured by this biological-infectious material?

☐ YES ☐ NO

Have you previously donated blood or undergone platelet apheresis?

☐ YES ☐ NO

Number of donations: ☐ 1 ☐ 2 ☐ 3 ☐ 4 ☐ 5 or more

Type of Sexual Relations:

Heterosexual: Sexual relations with the opposite gender.

Homosexual: Sexual relations with the same gender.

Bisexual: Sexual relations with both genders.

Total number of sexual partners in your lifetime:

☐ 1 ☐ 2 ☐ 3 ☐ 4 ☐ 5 or more

Have you received a blood transfusion or blood components?

☐ YES ☐ NO

Number of transfusions: ☐ 1 ☐ 2 ☐ 3 ☐ 4 ☐ 5 or more

Have you undergone previous surgeries or surgical interventions?

☐ YES ☐ NO

Number of surgeries: ☐ 1 ☐ 2 ☐ 3 ☐ 4 ☐ 5 or more

Have you had previous dental procedures?

☐ YES ☐ NO

Number of dental procedures: ☐ 1 ☐ 2 ☐ 3 ☐ 4 ☐ 5 or more

Have you received a solid organ transplant?

☐ YES ☐ NO

Have you been diagnosed with viral hepatitis (Hepatitis A, B, or C)?

☐ YES ☐ NO

If affirmative, have you received treatment for hepatitis?

☐ YES ☐ NO

Name of the treatment: ______________

Have you been diagnosed with any liver disease (other than viral hepatitis)?

☐ YES ☐ NO

If affirmative, please specify: ______________

Does anyone in your family have or have had Hepatitis C?

☐ YES ☐ NO

Do you currently live or have you lived with the affected individual?

☐ YES ☐ NO

Have you ever used drugs?

☐ YES ☐ NO

☐ Marijuana ☐ Cocaine ☐ Heroin ☐ Others: ______________

Do you regularly consume alcohol?

☐ YES ☐ NO

If affirmative, how often?

☐ Occasionally

☐ Weekly

☐ Daily

Do you have tattoos?

☐ YES ☐ NO

Number of tattoos: ☐ 1 ☐ 2 ☐ 3 ☐ 4 ☐ 5 or more

How many years ago did you get the first one? ______________

Have you had body piercings?

☐ YES ☐ NO

Number of piercings: ☐ 1 ☐ 2 ☐ 3 ☐ 4 ☐ 5 or more

How many years ago did you get the first one? ______________

Have you undergone needle therapy (acupuncture)?

☐ YES ☐ NO

Number of acupuncture treatments: ☐ 1 ☐ 2 ☐ 3 ☐ 4 ☐ 5 or more

How many years ago did you have the first one? ______________

Have you been in prison for more than 72 hours?

☐ YES ☐ NO

Have you been admitted to a psychiatric hospital?

☐ YES ☐ NO

Have you had an ingrown toenail removed?

☐ YES ☐ NO

Have you ever injured yourself with razors or blades that were not for exclusive personal use (e.g., at a barbershop or beauty salon)?

☐ YES ☐ NO

Have you undergone colposcopy?

☐ YES ☐ NO ☐ NOT APPLICABLE

How many times? ______________

Have you undergone endoscopy?

☐ YES ☐ NO

How many times? ______________

In gynecological examinations, has disposable equipment (e.g., vaginal speculum) been used?

☐ YES ☐ NO ☐ NOT APPLICABLE
