# Supplementary material for: Prevalence and Risk Factors of Occult HCV Infection in the Adult Population of Mexico City
Source: Viruses. 2025 Feb 8;17(2):236. doi: 10.3390/v17020236 (PMC11860181; doi:10.3390/v17020236)
Supplement: Supplementary file 1 [file viruses-17-00236-s001.zip › Table 1s.pdf]

Supplementary Table S1. Detailed genotyping results and patient characteristics

No.	Patient	Gender	Age (years)	Core/E1 sequencing	NCBI Genotyping tool	GenBank accession number	Anti-HCV CMIA*	Anti-HCV Rapid Test**	HCV-RNA plasma	HCV-RNA PMBC
1	50c	M	34	3a	3a	MZ997457	Negative	Negative	Negative	Positive
2	60c	M	37	2j	2j	MZ997458	Negative	Negative	Negative	Positive
3	65c	F	32	1b	1b	MZ997439	Negative	Negative	Negative	Positive
4	70c	F	39	1b	1b	MZ997438	Negative	Negative	Negative	Positive
5	160c	F	42	1b	1b	MZ997443	Negative	Negative	Negative	Positive
6	191c	M	31	1b	1b	MZ997434	Negative	Negative	Negative	Positive
7	192c	F	42	1b	1b	MZ997441	Negative	Negative	Negative	Positive
8	194c	M	48	1b	1b	MZ997435	Negative	Negative	Negative	Positive
9	198c	F	44	1a	1a	MZ997445	Negative	Negative	Negative	Positive
10	229c	F	32	1a	1a	MZ997451	Negative	Negative	Negative	Positive
11	230c	F	37	2b	2b	MZ997455	Negative	Negative	Negative	Positive
12	242c	F	35	1b	1b	MZ997453	Negative	Negative	Negative	Positive
13	248c	M	30	1b	1b	MZ997460	Negative	Negative	Negative	Positive
14	260c	M	19	3a	3a	MZ997446	Negative	Negative	Negative	Positive
15	267c	F	36	1a	1a	MZ997437	Negative	Negative	Negative	Positive
16	270c	M	31	1a	1a	MZ997456	Negative	Negative	Negative	Positive
17	279c	F	24	1b	1b	MZ997449	Negative	Negative	Negative	Positive
18	281c	M	21	1b	1b	MZ997450	Negative	Negative	Negative	Positive
19	282c	M	27	3a	3a	MZ997444	Negative	Negative	Negative	Positive
20	284c	F	24	1a	1a	MZ997440	Negative	Negative	Negative	Positive
21	295c	M	43	3a	3a	MZ997452	Negative	Negative	Negative	Positive
22	299c	M	68	1b	1b	MZ997454	Negative	Negative	Negative	Positive
23	308c	M	33	1b	1b	MZ997447	Negative	Negative	Negative	Positive
24	325c	M	42	1b	1b	MZ997442	Negative	Negative	Negative	Positive
25	356c	M	36	1b	1b	MZ997436	Negative	Negative	Negative	Positive
26	440c	M	34	1a	1a	MZ997448	Negative	Negative	Negative	Positive
27	474c	F	48	2b	2b	MZ997459	Negative	Negative	Negative	Positive

M: Male; F: Female; *CMIA: Chemiluminescent Microparticle ImmunoAssay; **Immunochromatographic rapid test; PBMC: Peripheral Blood Mononuclear Cell
